# Multi-modal monocular endoscopic depth and pose estimation with edge-guided self-supervision

**DOI:** 10.1007/s11548-026-03669-1

**Published:** 2026-05-10

**Authors:** Xinwei Ju, Rema Daher, Danail Stoyanov, Sophia Bano, Francisco Vasconcelos

**Affiliations:** Department of Computer Science, Hawkes Institute, University College London, 43–45 Foley Street, London, W1W 7TY UK

**Keywords:** Monocular depth estimation, Pose estimation, Self-supervised learning, Colonoscopy navigation

## Abstract

**Purpose:**

Monocular depth and pose estimation play an essential role in developing colonoscopy-assisted navigation systems. Accurate geometric understanding can reduce blind spots, lower the risk of missed or recurrent lesions, and prevent incomplete examinations. However, this task remains challenging due to texture-less surfaces, complex illumination, tissue deformation, and the scarcity of in-vivo datasets with reliable ground truth.

**Methods:**

We propose a self-supervised learning framework that leverages anatomical and illumination priors to guide geometric learning. The framework integrates two complementary cues: (1) edge maps, obtained from a learning-based detector trained to capture thin and high-frequency mucosal boundaries, and (2) luminance decomposition, produced through intrinsic image separation that isolates shading from reflectance. These modalities provide structural and photometric guidance to the pose and depth networks, with an edge-guided loss applied in a stage-wise refinement that enhances motion alignment while preserving depth consistency.

**Results:**

Experiments on phantom (C3VD) and real (EndoMapper) datasets demonstrate state-of-the-art performance in depth estimation and competitive accuracy in pose estimation. Ablation analyses further evaluate the influence of training domain, temporal sampling, and supervision type. Two practical findings emerge: Self-supervised training on real data outperforms supervised training on phantom data, highlighting the importance of domain realism; and dataset-specific frame-rate sampling is critical for generating effective training sequences.

**Conclusion:**

The proposed framework enhances geometric learning in endoscopic videos by incorporating structure- and illumination-aware cues, providing a robust foundation for reliable, marker-free colonoscopy navigation. The code and pretrained models are publicly available at: https://github.com/XinweiJu/PRISM.

**Supplementary Information:**

The online version contains supplementary material available at 10.1007/s11548-026-03669-1.

## Introduction

Gastrointestinal endoscopy is an essential procedure for detecting and treating cancer and other lesions in the digestive tract [1]. However, blind spots, limited visibility, and operator variability can result in missed polyps, incomplete examinations, or higher recurrence risk. Computer-assisted navigation can address these challenges by improving lesion detection and localization, supporting more reliable and effective screening.

Vision-based motion and depth estimation are key to such systems, enabling navigation without additional tracking devices or hardware modifications to a standard endoscope. Self-supervised learning has emerged as a robust framework for monocular depth and pose estimation [2–4]. These methods jointly train depth and pose networks via photometric consistency, inspiring adaptations to endoscopic imaging [5, 6]. However, performance remains challenging due to complex motion patterns, difficult illumination conditions.

Recent studies seek to exploit rather than suppress illumination cues [7, 8]: LightDepth [9] models light fall-off and specular reflection, while SHADeS [10] and IID-SfMLearner [11] decompose shading and reflectance. Meanwhile, geometric priors such as edges have shown promise, with DexiNed [12] adapted for mucosal fold detection [13, 14] and edge cues aiding polyp segmentation [15]. Together, these studies highlight the value of illumination and structural cues for robust depth and motion estimation, though their joint use as auxiliary inputs remains unexplored.

Furthermore, the lack of reliable ground truth pose and depth in real endoscopy videos makes model training and evaluation difficult. To compensate, synthetic and phantom datasets such as C3VD [16], SimCol [17], and EndoSLAM [18] are commonly used, but their substantial domain gap limits generalization. Consequently, supervised models trained on synthetic data often fail to transfer to real scenes, while self-supervised methods struggle under occlusions and illumination changes. Although domain adaptation techniques [19, 20] attempt to bridge this gap, the trade-offs between labeled synthetic and unlabeled real data remain underexplored.

To address these challenges, we propose **PRISM** (Pose Refinement with Intrinsic Shading and edge Maps), a stage-wise self-supervised framework that injects luminance cues into DepthNet and edge cues into PoseNet, followed by pose fine-tuning with an edge consistency loss while freezing depth. Specifically, colon fold edge maps from a DexiNed [12]-based **EdgeNet** trained on SegCol [14] provide crisp structural boundaries for motion estimation, while luminance maps from an intrinsic decomposition network (**LumNet**, following SHADeS [10]) help disambiguate shading from geometry. Training is performed in a stage-wise manner, where depth and pose networks are first jointly optimized under self-supervision, followed by a pose-only refinement stage guided by edge consistency while keeping depth fixed. This design yields more stable camera trajectories and sharper depth reconstructions in challenging endoscopic scenes. In particular, edge maps provide clearer structural boundaries for motion estimation, while luminance cues reduce ambiguity between lighting and surface geometry, remaining robust to specularities and low-texture endoscopic environments. Importantly, PRISM does not introduce new visual cues per se; instead, its contribution lies in a structured integration strategy (selective routing and stage-wise refinement), which stabilizes self-supervised learning under endoscopic illumination variability and texture scarcity. In addition, we also perform a range of comparative experiments to establish the best baseline methodology in terms of training data selection and supervision methodology. Our main contributions are as follows:

- We introduce PRISM, a multi-modal self-supervised framework that integrates luminance cues into DepthNet and edge cues into PoseNet, showing that this selective assignment improves geometric learning in endoscopy compared with using RGB alone.
- We design a stage-wise training strategy where edge maps are used as not only inputs but also supervisory signals through an edge consistency loss that fine-tunes PoseNet while freezing DepthNet, improving pose accuracy without degrading depth quality.
- We provide an analysis of training domain, temporal frame sampling, and weak supervision, offering new insights into how these factors influence depth and pose learning in endoscopy.

Our model achieves state-of-the-art depth estimation and comparable pose accuracy on phantom data, and demonstrating improved robustness to illumination and sharper depth contrast around fold edges in real data. Our systematic analysis further shows that: (a) training on real-world data yields better generalization than phantom or synthetic data, even on synthetic test sets; (b) optimal temporal sampling varies significantly across datasets and models; and (c) weak supervision (e.g., edge-guided loss) improves pose estimation without compromising depth accuracy.

## Methodology

**Fig. 1:**
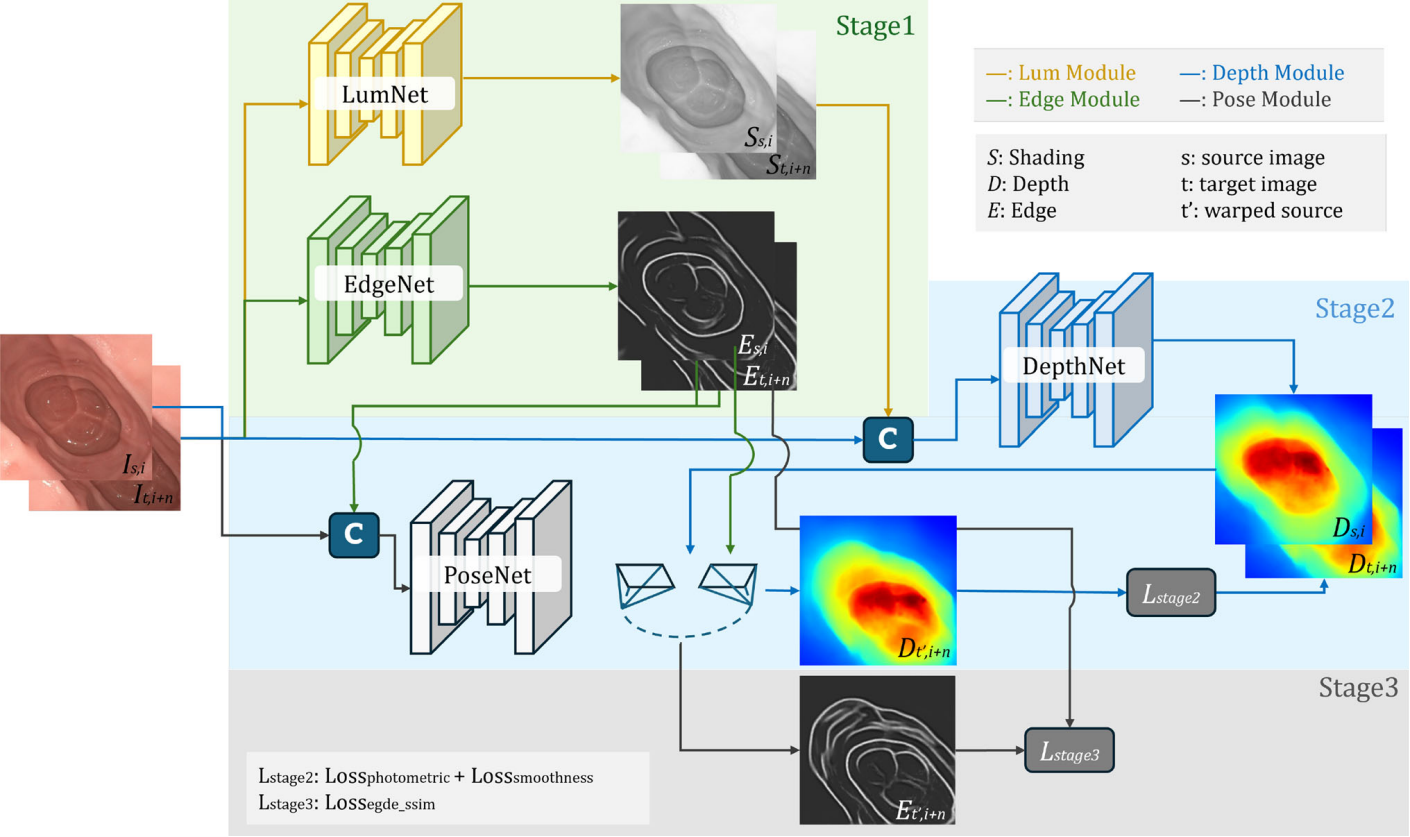
An overview of our proposed self-supervised monocular depth estimation framework. Our key contribution is to explicitly guide the DepthNet with priors from two parallel branches: a LumNet, inspired by IID-SfMLearner, to extract an illumination-aware luminance map ($L_t$), and an EdgeNet, based on DexiNed, to extract a high-fidelity structural edge map ($E_t$). The inputs to the DepthNet are the concatenation of the raw image $I_t$, the luminance map $L_t$, and the edge map $E_t$

**Fig. 2:**
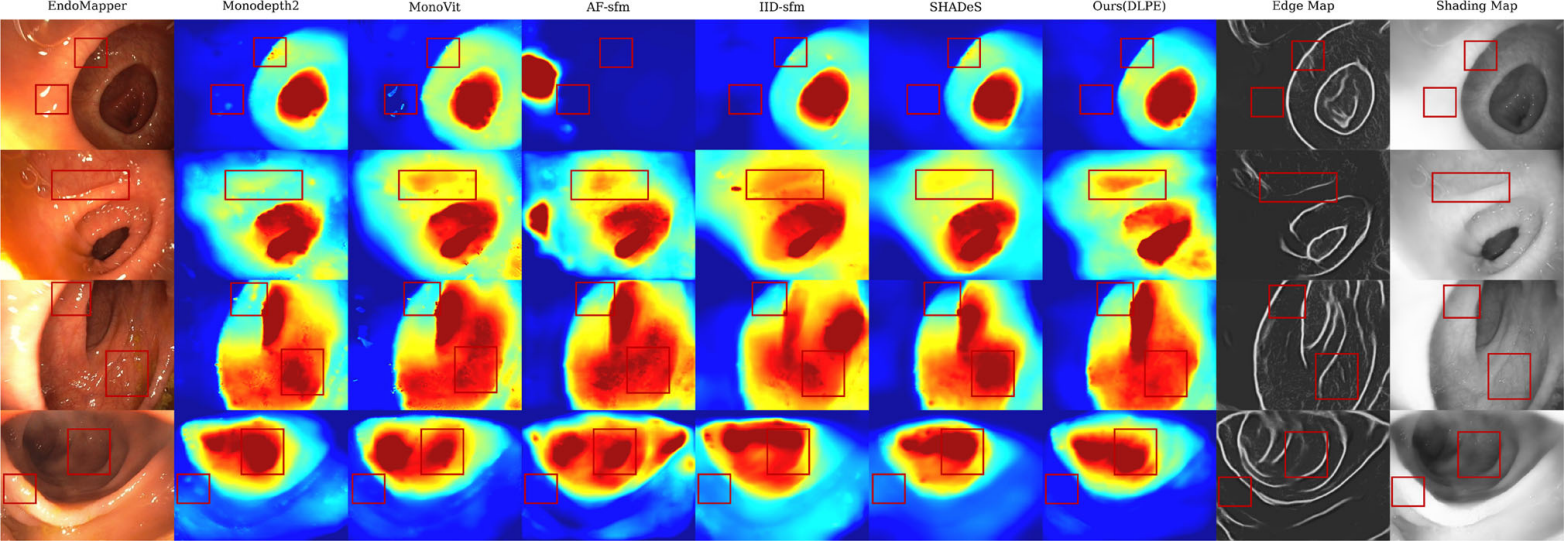
Qualitative evaluation of generalization on **real-world endoscopic video frames** with challenging improvements boxed in red

Without explicit structural cues, traditional self-supervised methods often yield unstable poses and blurred depth near fold boundaries (see Fig. 2). Our approach extends standard self-supervised monocular depth–pose frameworks [3] by introducing two auxiliary networks, LumNet and EdgeNet, that provide photometric and structural priors for endoscopic scenes to overcome the challenges caused by illumination variation, specular highlights, and low-texture surfaces. EdgeNet captures motion-related boundary features that aid pose estimation, while LumNet extracts illumination cues correlated with surface geometry to help disentangle lighting from shape, thereby stabilizing depth estimation. An overview of the full architecture is shown in Fig. 1, with each component detailed below.

### Network components and training strategy

Our framework consists of four networks: a luminance extractor (LumNet), an edge detector (EdgeNet), a depth estimator (DepthNet), and a pose estimator (PoseNet). LumNet, trained following SHADeS [10], extracts luminance maps by decoupling endoscopic images into luminance, albedo, and specularity components, which could suppresses specularities as well. EdgeNet, based on DexiNed [12], is trained from scratch on real colonoscopy data (SegCol [14]) labeled with fold segmentation maps in a fully supervised manner to produce high-fidelity fold edges. Rather than treating edges as view-invariant geometric primitives, we use them as view-dependent structural cues that highlight fold boundaries and curvature changes, whose appearance variation across viewpoints provides informative constraints for pose refinement. Following standard practice in [3], we utilize separate PoseNet and DepthNet networks for camera pose and depth estimation.

**Input assignment:** Unlike prior methods using only RGB frames, DepthNet take frames $(I_s, I_t)$ and luminance maps $(L_t, L_s)$ to account for depth-illumination correlation, while PoseNet predicts the 6-DoF transformation $\hat{T}_{t\rightarrow t^{\prime }}$ from paired RGB and edge inputs $(I_s, I_t, E_s, E_t)$, so that motion estimation is guided by fold-localizing edges. This selective routing allows illumination cues to regularize shape and edge cues to constrain motion, resulting in sharper depth and more stable trajectories.

**Stafe-wise training:** Training proceeds in three stages. First, LumNet and EdgeNet are pretrained and frozen. Second, PoseNet and DepthNet are jointly optimized using self-supervised photometric and smoothness losses extended with edge and luminance cues. Finally, we fine-tune PoseNet with an edge-aware geometric consistency loss while freezing DepthNet. We observed asymmetric effects of the two priors, with edges strengthening motion alignment whereas luminance stabilizing depth, so this stage-wise refinement ensures accurate motion without degrading depth quality.

### Self-supervised loss functions

DepthNet and PoseNet are jointly trained with a self-supervised objective enforcing geometric consistency between adjacent frames, with all losses computed at multiple scales following [3].

**Photometric reprojection **($L_{{\textbf {stage2}}}$): During stage 2, DepthNet and PoseNet are trained using photometric reprojection and gradient-guided smoothness losses:

1 $$\begin{aligned} L_{\text {stage2}} = \sum _{\sigma =0}^{3} \Big [&\lambda _{\text {photo}} \min _{t^{\prime }} pe(I_t^\sigma , I_{t^{\prime } \rightarrow t}^\sigma ) \nonumber \\ +&\lambda _{\text {smooth}} ( |\partial _x d_t^\sigma | e^{-|\partial _x I_t^\sigma |} + |\partial _y d_t^\sigma | e^{-|\partial _y I_t^\sigma |} ) \Big ] \end{aligned}$$

where σ indexes scaless; $I_t^\sigma$ and $d_t^\sigma$ denote the target image and inverse depth, while $\partial _x$, $\partial _y$ denote horizontal and vertical gradients, and $I_{t^{\prime } \rightarrow t}^\sigma$ is the source frame warped to the target using predicted depth and pose. The photometric error is defined as:

2 $$\begin{aligned} pe(I_a, I_b) = \tfrac{\alpha }{2}(1 - \text {SSIM}(I_a, I_b)) + (1 - \alpha )\Vert I_a - I_b\Vert _1 \end{aligned}$$

with α=0.85, taking the minimum over source views to mitigate occlusions. The smoothness term promotes spatial coherence while preserving edges. We set $\lambda _{\text {photo}}=1.0$ and $\lambda _{\text {smooth}}=0.1$.

**Edge-guided structural consistency **($L_{{\textbf {stage3}}}$): To refine geometric alignment, PoseNet is fine-tuned using an edge-guided structural consistency loss:

$$ L_{\text {stage3}} = L_{\text {stage2}} + \lambda _{\text {edge}} L_{\text {edge}}, \quad \lambda _{\text {edge}} = 1.0 $$

Stage 3 isolates edge supervision to address the asymmetry between depth and pose learning. For each source frame $t^{\prime }$ and each scale $\sigma \in \{0,1,2,3\}$, we extract its edge map $E_{t^{\prime }}^\sigma$ and warp it to the target view using the predicted depth $D_t^\sigma$ and relative pose $T_{t \rightarrow t^{\prime }}$. For each scale and source frame:

3 $$\begin{aligned} L_{\text {edge}} = \sum _{\sigma =0}^{3} \frac{1}{N^\sigma } \sum _x \left( 1 - \text {SSIM}(E_t^\sigma (x), E_{t^{\prime } \rightarrow t}^\sigma (x)) \right) \end{aligned}$$

where $E_{t^{\prime } \rightarrow t}^\sigma$ is the warped source edge map and $N^\sigma$ is the number of valid pixels. This loss shares the same warping transformation and SSIM formulation as the photometric term in $L_{\text {stage2}}$, but operates on edge maps to emphasize geometric alignment of object boundaries. While $L_{\text {stage2}}$ promotes appearance consistency across views, the edge-guided loss complements it by enforcing structural consistency, leading to depth and pose predictions that are both visually coherent and structurally precise across scales. This loss promotes the correct alignment of depth discontinuities when projected to 2D viewpoints, encouraging pose estimates that better align colon fold structures and reducing drift in low-texture regions.

## Experiments and results

### Experimental setup

**Datasets:** We employ multiple endoscopic datasets for training and evaluation. All self-supervised network components (LumNet, PoseNet, DepthNet) are trained on real sequences from the *Hyper-Kvasir* dataset [21], selecting clips with good bowel preparation (BBPS 2–3) and sampling at 25 fps, resulting in 16,976 frames; the edge detection network (EdgeNet) is trained on the *SegCol* subset [14] containing manually annotated fold contours with the same training and validation splits as the SegCol challenge. Quantitative evaluation are conducted on the *C3VD* phantom dataset [16], which provides pose and depth ground truth recorded at 30 fps. We report test results on four held-out trajectories (cecum_t4b, descending_t4a, sigmoid_t3b, transcending_t4b) following the split in [22]. For qualitative assessment, we use frames from sequences 1, 16, and 95 from the *EndoMapper* dataset [23] to show the performance on complete real endoscopy videos. We note that although C3VD provides dense synthetic ground truth, models trained on real Hyper-Kvasir data consistently yield better accuracy, even when evaluated on synthetic domains (Section “Ablation study”).

**Hyper-parameters:** Our framework is implemented in PyTorch and trained on a single NVIDIA A100-SXM GPU using Adam ($\beta _1\!=\!0.9$, $\beta _2\!=\!0.999$). The initial learning rate was $1\times 10^{-4}$ and reduced by ×0.1 after 15 epochs with a step scheduler. Inputs were undistorted according to camera intrinsics, cropped, and resized to 288×288; batch size was 12.

In Stage 1, EdgeNet was trained for 20 epochs; LumNet used SHADeS [10] pretrained weights (trained for 20 epochs). In Stages 2 and 3, PoseNet and DepthNet were trained for 20 epochs in each stage; in Stage 2 they were initialized with pre-trained weights from MonoDepth2 [3] on KITTI dataset.

**Evaluation metrics:** We adopt standard depth error metrics established by Eigen et al. [24] (Abs Rel, Sq Rel, RMSE, RMSE log). Additionally, we report the Mean Absolute Error (MAE) and Median Absolute Error (MedAE). For camera pose estimation, we follow the protocol from Rau et al. [17] and use the EVO toolkit [25] to report the Absolute Trajectory Error (ATE).

**Table 1:** Quantitative comparison of depth estimation on the C3VD test set for models trained with Hyper-Kvasir. Best results are in **bold**, second best are underlined

Model	RMSE ↓	LogRMSE ↓	MAE ↓	MedAE ↓	AbsRel ↓	SqRel ↓	ATE ↓
Monodepth2 [3]	6.3138	0.1841	4.3699	3.1279	0.1501	1.0168	2.7025
MonoViT [4]	6.8544	**0**.**1789**	4.6146	**3**.**0384**	**0**.**1473**	1.0958	2.4298
AF-SfM$_{\text {pretrained}}$ [7]	17.3395	0.5216	12.0918	8.2881	0.4126	7.2491	–
AF-SfM$_{\text {fine-tuned}}$ [7]	9.9636	0.2658	6.3069	3.4845	0.2165	3.7232	4.4841
IID-SfM$_{\text {pretrained}}$ [11]	17.6461	0.5269	12.3394	8.4590	0.4178	7.4915	–
IID-SfM$_{\text {fine-tuned}}$ [11]	8.6977	0.2552	5.6891	3.5772	0.1996	1.9452	3.1358
SHADeS [10]	7.1065	0.1845	4.7230	3.1341	0.1500	1.1437	**1**.**2533**
Ours	**6**.**1246**	0.1830	**4**.**3300**	3.2582	0.1475	**1**.**0010**	1.9077

### Comparison with state of the art

Our model is compared against both general-purpose [3, 4] and endoscopy-specific approaches [7, 10, 11], among which SHADeS [10] represents the most recent pose and depth estimation model fine-tuned for GI endoscopy. The methods in [7, 11] were originally trained on a different endoscopic domain (SCARED dataset [26]) and we evaluate both the original weights (pretrained) and a fine-tuned version on Hyper-Kvasir (HK). Daher et al. [10] is directly trained on Hyper-Kvasir (HK) so we report a single version of this model.

Table 1 shows that our method and MonoViT are the top performers in depth estimation on C3VD synthetic data. Our model performs best in metrics sensitive to large errors (e.g., RMSE), while MonoViT performs best in metrics without this characteristic (e.g., MedAE, MAE). However, MonoViT performs significantly worse than most other models on real data (Fig. 2), showing high sensitivity to light reflections, often producing undesirable artifacts. On the other hand, SHADeS also shows good robustness to reflections. Nonetheless, our method still produces sharper edges around colon folds (second row) and does not "hallucinate" lumen features in incorrect regions (third row). While our method performs well on both synthetic (C3VD) and real (EndoMapper) data, baselines show mixed results. Given the visual complexity of real endoscopy, we argue that strong performance on real data, despite lacking ground truth, should be prioritized.

We also include the edge map and shading map for real image at the right two columns in Fig. 2. In subtle mucosal folds in the upper-left region in the second row and the second row, and the lower boundary in the forth row, the clear prediction is detectable in the edge map and thus provide useful structural cues for depth estimation. However, in the first row, several spurious edges caused by bubble reflections appear in the edge map, yet they do not propagate to the final depth prediction.These observations indicate that the framework remains robust to moderate edge prediction noise while still benefiting from accurate structural cues.

For pose estimation, the ATE results in Table 1 indicate that SHADeS achieves the lowest ATE on phantom data, with our method ranking second. While structural priors can improve geometric consistency in depth prediction, the joint optimization of depth and pose may introduce a mild trade-off between the two objectives. In our framework, incorporating structural priors mainly benefits depth reconstruction and boundary preservation, which slightly alters the optimization balance for pose estimation. However, the introduction of the edge-aware loss in Stage 3 mitigates this effect by refining the pose estimation without modifying the depth predictions. This behavior is also supported by the ablation study in Table 2. Together, our method still achieves competitive pose accuracy while providing more reliable depth predictions and sharper structural details in endoscopic scenes.

### Ablation study

**Structural priors and edge-aware training:** In Table 2, we analyze the contribution of structural priors and edge-guided training strategies through a unified ablation study conducted under a consistent training and evaluation protocol. Among all variants, assigning luminance features to DepthNet and edge features to PoseNet (DLPE) achieves the lowest depth error and the most stable trajectories. In contrast, indiscriminate combinations of priors (e.g., DE, PL) or joint edge-loss training tend to degrade both depth and pose estimation; the complete set of ablation results covering all combinations is reported in the supplementary material Table 1. As a reference point, we start from a baseline corresponding to Monodepth2 self-supervised model trained on Hyper-Kvasir using RGB inputs only.

The results further reveal different roles of structural priors and edge-aware training. Introducing structural priors as input features improves depth estimation but slightly degrades pose accuracy, reflecting the coupled optimization between depth and camera motion. In contrast, the edge-aware loss mainly benefits pose estimation. As shown in Table 2, stage-wise fine-tuning of PoseNet with the edge-aware loss ($^\dagger$) significantly improves trajectory accuracy while preserving the depth performance obtained with DLPE, whereas joint end-to-end training slightly degrades both. Overall, these results demonstrate that (i) structural priors should be selectively assigned to each subnetwork, and (ii) stage-wise edge-aware refinement provides the best trade-off between depth and pose estimation.

**Table 2:** Quantitative comparison between baseline Monodepth2 [3] and PRISM with DLPE settings of depth and pose estimation on the C3VD test set for models trained with Hyper-Kvasir.

Model	RMSE ↓	LogRMSE ↓	MAE ↓	MedAE ↓	AbsRel ↓	SqRel ↓	ATE ↓
Monodepth2 [3]	6.3138	0.1841	4.3699	3.1279	0.1501	1.0168	2.7025
Monodepth2 [3] + EdgeLoss (joint)	6.8043	0.1991	4.9267	3.7038	0.1681	1.2604	**1**.**6293**
DLPE	**6**.**1246**	0.1830	**4**.**3300**	3.2582	0.1475	**1**.**0010**	2.5101
DLPE + EdgeLoss (joint)	6.5946	**0**.**1761**	4.4031	**2**.**9848**	**0**.**1388**	1.0138	2.5858
DLPE + EdgeLoss (stage$^\dagger$)	**6**.**1246**	0.1830	**4**.**3300**	3.2582	0.1475	**1**.**0010**	1.9077

Best results are in **bold**, second best are underlined

$^\dagger$Stage-wise: fine-tuning PoseNet only, with DepthNet frozen

**Impact of training domain, frame interval, and supervision:** Beyond model configuration, we further investigate how dataset domain and temporal sampling influence self-supervised learning stability. To this end, we conduct the analysis under a fixed PRISM with DLPE setting, and systematically vary the training domain (C3VD vs. Hyper-Kvasir), temporal interval, and supervision mode. For clarity, Table 3 reports representative settings: the base interval on C3VD (interval=1), the best-performing interval on C3VD (interval=30), the supervised variant on C3VD at the same interval, and the best-performing configuration on Hyper-Kvasir (interval=1). This table focuses on data-centric factors (domain, temporal interval, and supervision mode) under a fixed PRISM configuration to analyze training stability trends.

**Fig. 3:**
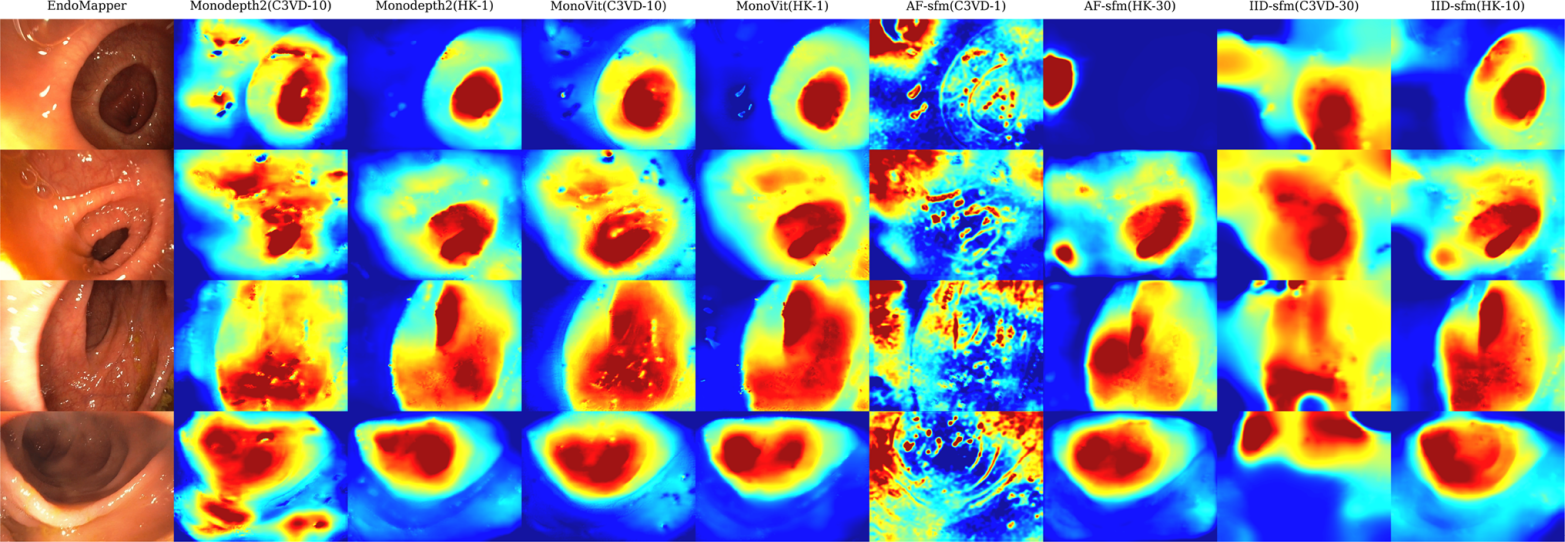
Qualitative generalization performance of models trained with Hyper-Kvasir on EndoMapper real dataset. This evaluation highlights model robustness to real-world challenges like specular reflections and complex tissue folds, where Hyper-Kvasir-trained models consistently show an advantage

The results show that training on real Hyper-Kvasir data yields the best generalization on C3VD, demonstrating the benefit of real-domain diversity even when evaluated on phantom data. On C3VD, larger temporal intervals provide stronger motion cues for learning, whereas incorporating supervised depth on C3VD does not lead to further improvements, likely due to noise and incompleteness in the available ground truth. For completeness, a separate supervised versus self-supervised comparison for Monodepth2 is reported in the supplementary material Table 3, showing similar trends in favor of self-supervised training. We also evaluate other baseline families across datasets and temporal intervals and observe largely consistent trends; the complete quantitative results are provided in the supplementary material Table 4, and qualitative EndoMapper examples are shown in Fig. 3. Overall, self-supervised training on real data consistently leads to more robust accuracy and generalization across datasets and temporal sampling settings.

**Table 3:** Comparison of depth estimation across datasets, temporal intervals, and supervision modes using PRISM with DLPE settings

Model	Training Set	Interval	RMSE ↓	LogRMSE ↓	MAE ↓	MedAE ↓	AbsRel ↓	SqRel ↓
**PRISM**	C3VD	1	14.4647	0.4165	9.7689	6.3846	0.3221	4.7946
	C3VD	30	7.7530	0.2460	5.4256	3.9831	0.1972	1.6139
	C3VD_supervised	30	8.2836	0.2523	5.6774	3.8749	0.1976	1.7205
	Hyper-phasir	1	**6**.**1246**	**0**.**1830**	**4**.**3300**	**3**.**2582**	**0**.**1475**	**1**.**0010**

Bold indicates the best performance for each metric

## Discussion

Our proposed model, which selectively integrates luminance and edge cues, achieves the most balanced performance across both synthetic and real domains. On the C3VD synthetic dataset, it outperforms most baselines in metrics sensitive to large reconstruction errors, while remaining competitive with MonoViT on median-based metrics. On the EndoMapper real dataset, qualitative comparisons reveal sharper structural boundaries around colon folds, fewer reflection artifacts, and more anatomically consistent lumen representation, indicating better robustness to illumination changes and texture ambiguity. Overall, these results suggest that luminance cues stabilize geometric learning and edge cues constrain motion, jointly leading to more reliable depth and pose estimation.

The ablation study confirms that both edge and luminance features enhance depth estimation, albeit at the cost of reduced pose estimation accuracy. We achieve state-of-the-art level pose estimation only through an Edge-Aware refinement stage. Notably, luminance features are best exploited by the DepthNet encoder, while edge features are most beneficial in the PoseNet encoder, though both feature types jointly contribute to improved depth estimation in the joint training stage.

Across all experiments, we find significant differences between phantom (C3VD) and real (Hyper-Kvasir) datasets in both training and testing. Models performing well on C3VD often fail to generalize to real data (e.g., MonoVIT), likely because real data contains richer visual features and more complex motion patterns. We also identify video sub-sampling as a key factor influencing performance, with its optimal setting depending heavily on the motion characteristics of the training data. Taken together, these results show that depth and pose estimation performance is strongly influenced by dataset realism and temporal sampling strategies. Rather than being a limitation, this dependency is explicitly analyzed in our study through controlled experiments. Importantly, under matched training data and temporal sampling conditions, our method consistently outperforms competing approaches, demonstrating its effectiveness under fair and comparable evaluation settings.

Our framework leverages a pre-trained EdgeNet to extract structural priors that guide both depth and pose estimation. Importantly, the edges predicted by EdgeNet are used as structural priors rather than direct supervision for depth prediction. The primary supervision still comes from the photometric reprojection loss, which enforces multi-view geometric consistency. As a result, occasional errors or missed detections in the edge maps do not directly corrupt the depth or pose estimates. In practice, the detected edges tend to align with stable anatomical boundaries in endoscopic scenes, such as folds and lumen structures, which provide useful structural signals across datasets. Nevertheless, exploring adaptive or jointly learned edge representations could further improve robustness and generalization, which we consider an interesting direction for future work.

## Conclusion

Our proposed model, which integrates luminance and edge features, delivers more robust depth estimation than most baseline methods. Edge guidance improves motion alignment, while luminance enhances geometric consistency, and their selective use through stage-wise optimization achieves an effective balance between depth and pose accuracy. Across datasets, we further observe that variations in domain characteristics and temporal sampling strongly influence model performance, underscoring the importance of data realism and motion dynamics in self-supervised learning. Future research could explore adaptive fusion strategies and domain adaptation techniques to further improve cross-domain generalization.

Although improvements have been obtained in terms of depth and pose accuracy, we note that further progress in this research area will require additional clinical validation beyond the currently available public benchmarks (Hyper-kvazir, C3VD, Endomapper). Future work should involve medical teams more directly to assess clinical utility and workflow integration of depth and pose models.

## Supplementary Information

Below is the link to the electronic supplementary material.

Supplementary file 1 (pdf 27186 KB)
