# Supplementary material for: Multi-modal monocular endoscopic depth and pose estimation with edge-guided self-supervision
Source: Int J Comput Assist Radiol Surg. 2026 May 10;21(6):1207–15. doi: 10.1007/s11548-026-03669-1 (PMC13451345; doi:10.1007/s11548-026-03669-1)
Supplement: Supplementary file 1 [file 11548_2026_3669_MOESM1_ESM.pdf]

# Multi-Modal Monocular Endoscopic Depth and Pose Estimation with Edge-Guided Self-Supervision

Xinwei Ju[1*], Rema Daher[1], Danail Stoyanov[1], Sophia Bano[1], Francisco Vasconcelos[1]

[1]Hawkes Institute, Department of Computer Science, University College London, 43–45 Foley Street, London, W1W 7TY, UK.

*Corresponding author(s). E-mail(s): xinwei.ju.22@ucl.ac.uk;

## 1 Luminance and Edge Features

We evaluate the role of luminance and anatomy edge features by providing them as input to DepthNet and PoseNet in different combinations. Table 1 shows depth estimation ablation results on C3VD, where the `DLPE` configuration denotes our proposed model. Best results are obtained by providing Luminance and Edge features to DepthNet and PoseNet respectively (`DLPE`) or by providing luminance features to both models (`DLPL`). Some variants either degrade performance or show no clear benefit, indicating that indiscriminate assignment is ineffective. We also show qualitative

**Table 1** Quantitative comparison of depth estimation with models trained on Hyper-Kvasir and tested on the C3VD test set. Best results are in **bold** and second best results are in underlined.

| Models | Pose Edge | Pose Lum | Depth Edge | Depth Lum | RMSE ↓ | Log RMSE ↓ | MAE ↓ | MedAE ↓ | Abs Rel ↓ | Sq Rel ↓ |
|---|---|---|---|---|---|---|---|---|---|---|
| Baseline | – | – | – | – | 6.2258 | 0.1845 | <u>4.3176</u> | 3.0993 | 0.1523 | 1.0360 |
| `PE` | ✓ | – | – | – | 6.2501 | <u>0.1824</u> | 4.3260 | <u>2.9790</u> | 0.1505 | 1.0610 |
| `PL` | – | ✓ | – | – | 6.7658 | 0.2072 | 4.8068 | 3.5452 | 0.1719 | 1.2877 |
| `DE` | – | – | ✓ | – | 6.6625 | 0.2111 | 4.7625 | 3.4991 | 0.1755 | 1.3095 |
| `DL` | – | – | – | ✓ | 6.7540 | 0.2005 | 4.7099 | 3.4705 | 0.1649 | 1.2652 |
| `DEPL` | – | ✓ | ✓ | – | 6.9001 | 0.2105 | 4.9384 | 3.5381 | 0.1739 | 1.3404 |
| `DLPE` | ✓ | – | – | ✓ | **6.1246** | 0.1830 | 4.3300 | 3.2582 | <u>0.1475</u> | <u>1.0010</u> |
| `DEPE` | ✓ | – | ✓ | – | 6.5529 | 0.1943 | 4.5099 | 3.0780 | 0.1558 | 1.1699 |
| `DLPL` | – | ✓ | – | ✓ | <u>6.2562</u> | **0.1688** | **4.2677** | **2.9406** | **0.1341** | **0.9384** |

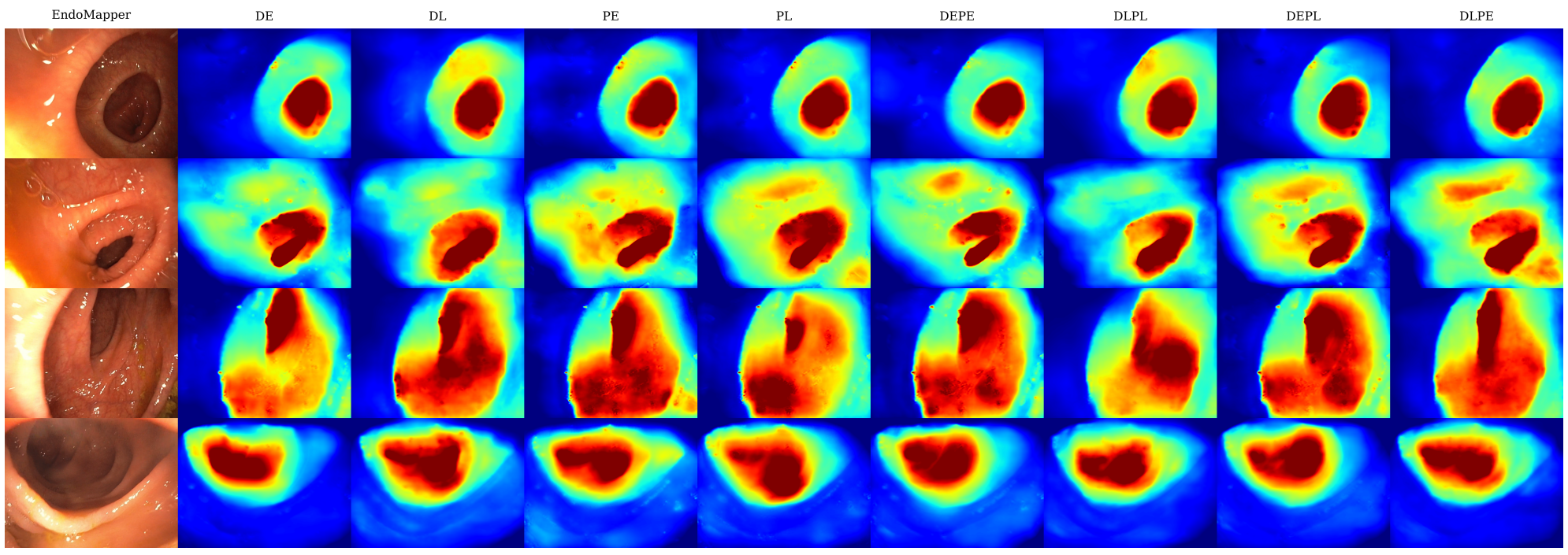


**Fig. 1** Qualitative comparison of ablation variants on four subsequences from the EndoMapper dataset. Each row shows a sample frame and the corresponding predicted depth maps from different prior configurations.

ablation results on EndoMapper Real data in Fig. 1, which shows `DLPE` to outperform `DLPL` in terms of fewer light artifacts (first row), more well defined edge contours (second row), and correct lumen location (third row). We also note some performance differences between quantitative results on phantom (C3VD) and real (EndoMapper). By prioritising results on Real-Data, we conclude `DLPE` is overall a more robust configuration than `DLPL`.

## 2 Refinement with Edge-Aware Loss

We assess the effect of the Edge-aware loss in Table 2. For the baseline (Monodepth) and the best performing configurations from Sec. 1 (DLPE, DLPL) we compare the following:

- No Edge-aware loss
- Joint training of PoseNet and DepthNet with Edge-aware loss;
- Refining PoseNet alone with Edge-aware loss in stage 3 (our proposed strategy);

In Table 1, DLPL denotes a model variant that does not inject edge features into either DepthNet or PoseNet. In Table 2, the "Edge Loss" column refers to the use of the edge-guided loss as an auxiliary training objective. Therefore, DLPL + Edge Loss indicates that the model is trained with the edge-guided loss while still not using edge features as inputs to either network. Stage 3 consistently improves camera trajectory reconstruction (lower ATE), however, it degrades depth estimation results if DepthNet is jointly trained in stage 3. We therefore conclude the best strategy is to freeze DepthNet in stage 3 so that we can obtain improvements in trajectory reconstruction without degrading depth estimation. Figure 2 further highlights visually the improvement in trajectories when refining stage 3 with Edge-aware loss.

## 3 Training Data Domain, Sampling, and Supervision

**Domain and Sampling:** We further assess the influence of training data domain and sampling as critical factors for good performance that have been overlooked in

**Table 2** Depth and pose estimation results of models trained with Hyper-Kvasir and tested on C3VD test set with different combinations of structural priors and edge-guided loss. Best configuration per model group is in **bold**.

| Model | Edge Loss | Pose Estimation | | | Depth Estimation | | | | | |
|---|---|---|---|---|---|---|---|---|---|---|
| | | ATE ↓ | RTE ↓ | ROT | RMSE ↓ | Log RMSE ↓ | MAE ↓ | MedAE ↓ | AbsRel ↓ | SqRel ↓ |
| Baseline | – | 2.7025 | 0.2004 | 0.1250 | 6.3138 | 0.1841 | 4.3699 | 3.1279 | 0.1501 | 1.0168 |
| | ✓ | 1.6293 | 0.2121 | 0.1286 | 6.8043 | 0.1991 | 4.9267 | 3.7038 | 0.1681 | 1.2604 |
| DLPE | – | 2.5101 | 0.2005 | 0.1408 | 6.1246 | 0.1830 | 4.3300 | 3.2582 | 0.1475 | 1.0010 |
| | ✓ | 2.5858 | 0.2015 | 0.1412 | 6.5946 | 0.1761 | 4.4031 | 2.9848 | 0.1388 | 1.0138 |
| | ✓† | 1.9077 | 0.2087 | 0.1496 | 6.1246 | 0.1830 | 4.3300 | 3.2582 | 0.1475 | 1.0010 |
| DLPL | – | 3.1202 | 0.1953 | 0.1464 | 6.2562 | 0.1688 | 4.2677 | 2.9406 | 0.1341 | 0.9384 |
| | ✓ | 2.1662 | 0.2050 | 0.1453 | 6.3535 | 0.1747 | 4.4400 | 3.2086 | 0.1426 | 1.0030 |
| | ✓† | 2.6802 | 0.2047 | 0.1458 | 6.2562 | 0.1688 | 4.2677 | 2.9406 | 0.1341 | 0.9384 |

✓ indicates that the edge-guided loss is jointly applied to both DepthNet and PoseNet during end-to-end training. ✓† denotes stage-wise training, where the edge-guided loss is only introduced when fine-tuning PoseNet, with DepthNet frozen. Red-shaded cells indicate that the metric value is worse compared to the baseline ("–") in the same block, while green-shaded cells indicate improvement.

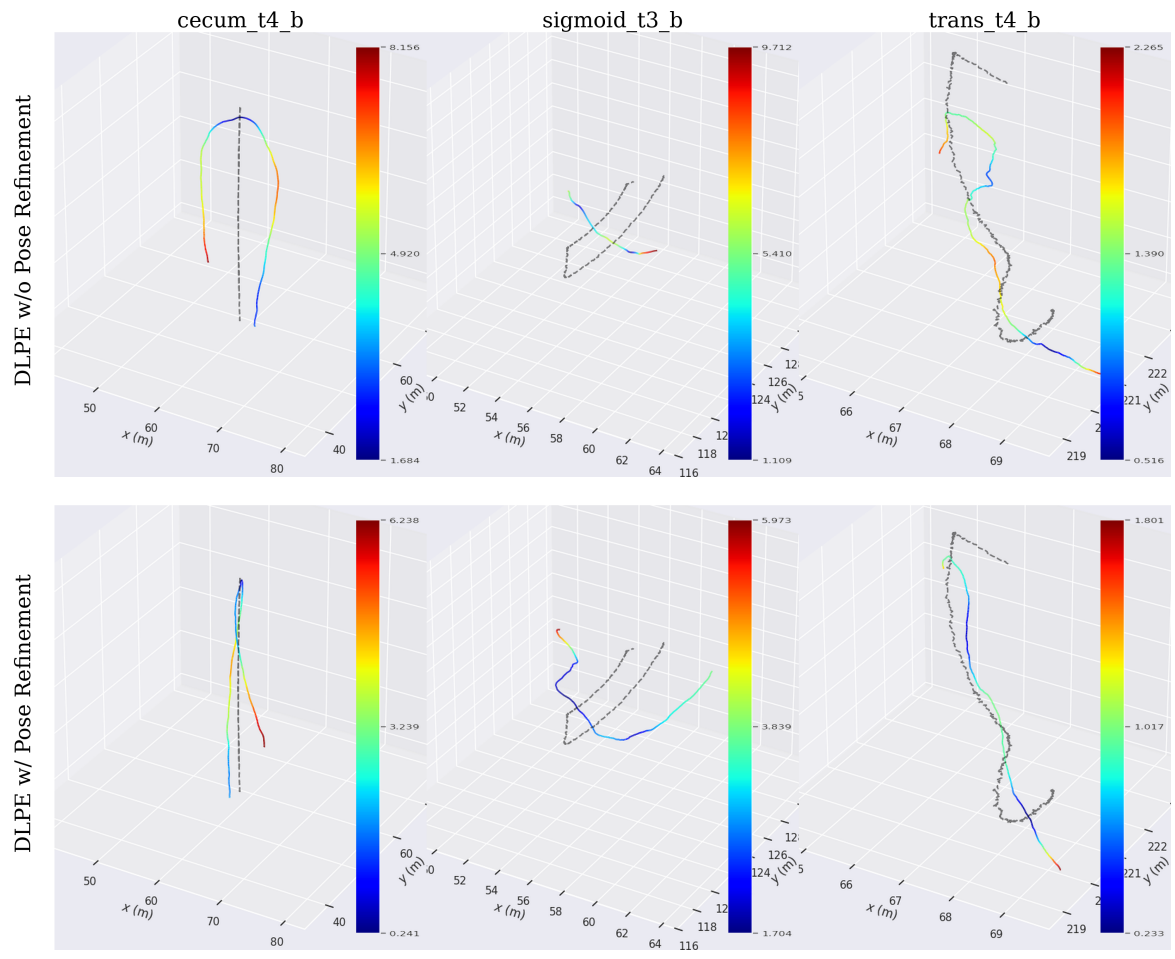


**Fig. 2** Qualitative trajectory comparison under modality-only vs. modality + edge-guided SSIM training on `cecum_t4b`, `sigmoid_t3b` and `trans_t4b`.

prior work. Table 4 reports depth accuracy on C3VD using models trained on either C3VD or Hyper-Kvasir. We also evaluate the effect of subsampling video frame rates during training. Surprisingly, training on Hyper-Kvasir (real data) consistently outperforms training on C3VD (phantom), even though evaluation is performed on the C3VD test data, underscoring the value of real data. Beyond visual domain differences, the datasets vary in endoscope motion; C3VD features slower, smoother movements, which may be too subtle for PoseNet to effectively learn motion cues. As shown in

**Table 3** Comparison between self-supervised and supervised Monodepth2 [1] models on C3VD (interval = 10). Supervised model adds scale-consistent depth loss with a weight of 0.5. The better one is marked in **Bold**

| Model | RMSE ↓ | LogRMSE ↓ | MAE ↓ | MedAE ↓ | AbsRel ↓ | SqRel ↓ |
|---|---|---|---|---|---|---|
| Self-supervised | **7.5960** | **0.2215** | **5.0917** | **3.4709** | **0.1732** | **1.4608** |
| Supervised (+GT) | 8.2805 | 0.2443 | 5.6020 | 3.7030 | 0.1921 | 1.7754 |

Table 4, effective training on C3VD requires subsampling by a factor of 10, while no subsampling is required for Hyper-Kvasir training. Even with optimal subsampling for both, Hyper-Kvasir training yields significantly better results.

**Supervision:** Since C3VD contains reliable depth ground truth, we also explore a supervised training strategy using the optimal subsampling rate (10). Specifically, we augment the baseline with a supervised loss, using optimally tuned weights:

$$L_{\text{stage2}} = L_{\text{photo}} + 0.1 \times L_{\text{smooth}} + 0.5 \times L_{\text{sup}}, \quad (1)$$

where $L_{\text{sup}}$ is the L1 distance between predicted and ground truth depth.

Surprisingly, adding supervised depth loss degrades performance across all metrics compared to the purely self-supervised baseline as shown in Table 3. Although the weight on $L_{\text{sup}}$ is moderate, it can dominate in low-texture regions with weak photometric gradients, leading to memorization of noisy targets rather than learning multi-view consistency. This is partly due to the limitations of C3VD's ground truth, obtained via structured light scanning and trajectory alignment. Although scale-consistent, the depth maps still contain noise and lack valid information in occluded regions.

**Summary:** Overall, Pose and Depth estimation perform best when trained self-supervised on real data. Despite being the best available endoscopy dataset with depth and pose labels, C3VD has notable limitations due to its phantom-based nature.

## References


[1] Godard, C., Mac Aodha, O., Firman, M., Brostow, G.J.: Digging into self-supervised monocular depth estimation. In: Proceedings of the IEEE/CVF International Conference on Computer Vision, pp. 3828–3838 (2019). https://doi.org/10.1109/ICCV.2019.00393

[2] Zhao, C., Zhang, Y., Poggi, M., Tosi, F., Guo, X., Zhu, Z., Huang, G., Tang, Y., Mattoccia, S.: Monovit: Self-supervised monocular depth estimation with a vision transformer. In: 2022 International Conference on 3D Vision (3DV), pp. 668–678 (2022). https://doi.org/10.1109/3DV57658.2022.00077 . IEEE

[3] Shao, S., Pei, Z., Chen, W., Zhu, W., Wu, X., Sun, D., Zhang, B.: Self-supervised monocular depth and ego-motion estimation in endoscopy: Appearance flow to the

**Table 4** Quantitative depth estimation results across models, datasets, and frame intervals. All models are trained on either C3VD or Hyper-Kvasir and always tested on C3VD test data.

| Model | Training Dataset | Interval | RMSE ↓ | RMSE log ↓ | MAE ↓ | MedAE ↓ | Abs Rel ↓ | Sq Rel ↓ | $\delta < 1.25$ ↑ | $\delta < 1.25^2$ ↑ | $\delta < 1.25^3$ ↑ |
|---|---|---|---|---|---|---|---|---|---|---|---|
| Monodepth2 [1] | C3VD | 1 | 15.1642 | 0.4196 | 10.1482 | 6.3000 | 0.3047 | 4.7856 | 0.4516 | 0.7353 | 0.8783 |
| | | 10 | **7.5960** | **0.2215** | **5.0917** | **3.4709** | **0.1732** | **1.4608** | **0.7161** | **0.9253** | **0.9827** |
| | | 20 | 7.7644 | 0.2473 | 5.4523 | 3.9334 | 0.2002 | 1.7340 | 0.6668 | 0.8976 | 0.9725 |
| | | 30 | 7.7282 | 0.2403 | 5.2392 | 3.6111 | 0.1878 | 1.6480 | 0.6919 | 0.8956 | 0.9696 |
| | Hyper-Kvasir | 1 | **6.3138** | **0.1841** | **4.3699** | **3.1279** | **0.1501** | **1.0168** | **0.7754** | **0.9720** | **0.9956** |
| | | 10 | 40.5039 | 1.3728 | 32.2524 | 26.0333 | 1.5865 | 93.1729 | 0.1331 | 0.2595 | 0.3706 |
| | | 20 | 17.4583 | 0.5306 | 12.2546 | 8.6783 | 0.4248 | 7.4185 | 0.3636 | 0.6225 | 0.7901 |
| | | 30 | 17.4583 | 0.5306 | 12.2546 | 8.6783 | 0.4248 | 7.4185 | 0.3636 | 0.6225 | 0.7901 |
| MonoViT [2] | C3VD | 1 | 15.9045 | 0.4543 | 10.7178 | 6.7776 | 0.3394 | 5.5865 | 0.4373 | 0.7044 | 0.8527 |
| | | 10 | **6.2371** | **0.1625** | **3.8398** | **2.3075** | **0.1203** | **0.8766** | **0.8427** | **0.9753** | **0.9929** |
| | | 20 | 6.5954 | 0.1797 | 4.1504 | 2.4064 | 0.1350 | 1.0694 | 0.8139 | 0.9442 | 0.9902 |
| | | 30 | 6.4839 | 0.1804 | 4.0457 | 2.4405 | 0.1397 | 1.1434 | 0.8137 | 0.9415 | 0.9900 |
| | Hyper-Kvasir | 1 | **6.8544** | 0.1789 | **4.6146** | **3.0384** | **0.1473** | **1.0958** | **0.7884** | **0.9788** | **0.9982** |
| | | 10 | 30.9196 | **1.1751** | 24.6074 | 21.0623 | 0.9914 | 35.7517 | 0.1471 | 0.2997 | 0.4428 |
| | | 20 | 30.8911 | 1.0684 | 23.9879 | 19.5039 | 1.1202 | 51.3599 | 0.1968 | 0.3551 | 0.4864 |
| | | 30 | 34.5043 | 1.1523 | 26.7642 | 21.4713 | 1.2017 | 58.8600 | 0.1739 | 0.3336 | 0.4751 |
| AF-SfMLearner [3] | C3VD | 1 | **16.8063** | **0.4929** | **11.3895** | **7.4673** | **0.3737** | **6.6151** | **0.4128** | **0.6786** | **0.8312** |
| | | 10 | 17.1426 | 0.5008 | 11.7025 | 7.5752 | 0.3847 | 6.8968 | 0.3962 | 0.6698 | 0.8239 |
| | | 20 | 17.2341 | 0.5128 | 11.8870 | 7.8705 | 0.3918 | 6.9788 | 0.3735 | 0.6483 | 0.8199 |
| | | 30 | 17.4512 | 0.5248 | 12.3266 | 8.5111 | 0.4245 | 7.6151 | 0.3596 | 0.6349 | 0.8001 |
| | Hyper-Kvasir | 1 | 9.9727 | **0.2660** | 6.3114 | **3.4862** | **0.2166** | 3.7265 | **0.6719** | **0.8900** | 0.9591 |
| | | 10 | 11.5691 | 0.3057 | 7.1733 | 4.1365 | 0.2540 | 4.5569 | 0.6146 | 0.8672 | 0.9556 |
| | | 20 | 10.1226 | 0.2833 | 6.4429 | 3.8149 | 0.2223 | **2.7322** | 0.6576 | 0.8663 | 0.9534 |
| | | 30 | **9.9544** | 0.2740 | **6.2987** | 3.8747 | 0.2240 | 3.2430 | 0.6713 | 0.8829 | **0.9625** |
| IID-SfMLearner [4] | C3VD | 1 | 14.3235 | 0.3844 | 9.3650 | **5.4199** | **0.2766** | 4.1887 | **0.4989** | 0.7773 | 0.9040 |
| | | 10 | 13.8868 | **0.3823** | **9.1635** | 5.5565 | 0.2827 | **4.1115** | 0.4973 | **0.7853** | **0.9094** |
| | | 20 | 14.3038 | 0.3988 | 9.5545 | 6.0755 | 0.2996 | 4.4211 | 0.4753 | 0.7667 | 0.9006 |
| | | 30 | **13.7964** | 0.3858 | 9.1892 | 5.8469 | 0.2967 | 4.1495 | 0.4856 | 0.7791 | 0.9056 |
| | Hyper-Kvasir | 1 | 8.6977 | 0.2552 | 5.6891 | 3.5772 | 0.1996 | 1.9452 | 0.6656 | 0.8773 | 0.9624 |
| | | 10 | **8.4079** | **0.2319** | **5.3326** | **3.2531** | 0.1829 | **1.6545** | **0.7237** | 0.9101 | 0.9767 |
| | | 20 | 8.8540 | 0.2357 | 5.6743 | 3.5312 | **0.1821** | 1.7427 | 0.7007 | **0.9103** | **0.9807** |
| | | 30 | 9.1189 | 0.2465 | 5.8221 | 3.4976 | 0.1900 | 1.9096 | 0.6988 | 0.8968 | 0.9701 |

rescue. Medical image analysis **77**, 102338 (2022) https://doi.org/10.1016/j.media.2021.102338

[4] Li, B., Liu, B., Zhu, M., Luo, X., Zhou, F.: Image intrinsic-based unsupervised monocular depth estimation in endoscopy. IEEE Journal of Biomedical and Health Informatics (2024) https://doi.org/10.1109/JBHI.2024.3400804
